# Neural Hyperactivity of the Central Auditory System in Response to Peripheral Damage

**DOI:** 10.1155/2016/2162105

**Published:** 2016-01-10

**Authors:** Yi Zhao, Qiang Song, Xinyi Li, Chunyan Li

**Affiliations:** Department of Otolaryngology, Shanghai Jiao Tong University Affiliated Sixth People's Hospital, Shanghai 200233, China

## Abstract

It is increasingly appreciated that cochlear pathology is accompanied by adaptive responses in the central auditory system. The cause of cochlear pathology varies widely, and it seems that few commonalities can be drawn. In fact, despite intricate internal neuroplasticity and diverse external symptoms, several classical injury models provide a feasible path to locate responses to different peripheral cochlear lesions. In these cases, hair cell damage may lead to considerable hyperactivity in the central auditory pathways, mediated by a reduction in inhibition, which may underlie some clinical symptoms associated with hearing loss, such as tinnitus. Homeostatic plasticity, the most discussed and acknowledged mechanism in recent years, is most likely responsible for excited central activity following cochlear damage.

## 1. Introduction

The mammalian auditory system falls broadly into two pieces, the auditory periphery and the central auditory system. The auditory periphery, which comprises the sound receptors, performs an acoustoelectric transformation. The electrical signals are then sent to the central auditory system for further processing, and eventually, the sensation of sound occurs. In pathological conditions, hearing impairment usually develops as a result of receptor dysfunction. For example, the great majority of acquired sensorineural hearing loss is caused by damage to hair cells in the cochlea. Risk factors for this kind of hearing loss include administration of ototoxic drugs, aging, and overexposure to noise. In fact, among the various manifestations of hearing pathologies confined to the auditory periphery, hearing loss is the most common one.

Pathology of the cochlea as a cause of hearing loss has been investigated comprehensively in recent years. However, the role of the central auditory system in hearing loss is still not fully understood. Except in rare cases where the brain and cochlea are both impaired by certain agents, brain changes are considered to be a response to an altered input from cochlea, rather than being directly caused by chemical or environmental factors. These neural changes in the central auditory system, that is, auditory neuroplasticity, have been observed in a broad range of brain behaviors during development and maturation [1–3]. Auditory neuroplasticity can also be observed after hearing loss as a kind of reactive adaption [4]. One of the most common topics of focus in this field concerns the mechanism(s) of tinnitus, that is, perception of sound in the absence of any stimulus, a condition that may develop following sensorineural damage to the auditory structures. Because tinnitus is often associated with a variety of hearing pathologies, attention has been paid to the link between them.

It has long been appreciated that tinnitus appears to persist in the patients diagnosed with acoustic neuroma and in cases where the auditory nerve is transected. Indeed, recent animal experiments have confirmed this by showing an overexcited state in the auditory brainstem after acoustic overstimulation, regarded as a behavioral sign of tinnitus [5, 6]. However, tinnitus has also been found independent of cochlear activity [7, 8]. Although little consensus has been achieved, it is considered that neural plasticity plays a critical role in development of tinnitus [9, 10].

The aim of this review is to assess how peripheral pathologies are associated with different damage agents and especially how pathologies of sensory cells influence neurons in the central auditory pathways. Responses to different damage agents are compared, and some commonalities and correlations among various hearing pathologies are discussed.

## 2. Influence of the Cochlea as a Whole

### 2.1. Cochlear Ablation

Important parallels have been drawn between this topic and the current state of research on neuroplasticity in the visual system. Vision and hearing are two major human sensations and share many similar structures and functions. Visual deprivation brings about changes in response properties of neurons in the visual circuits. For example, when one eye is covered, the spiking responses of visual cortical neurons are shifted in favor of the untreated eye, and visual acuity in the blocked eye is reduced [11–13]. Central auditory pathways may also be subject to modification as a result of alterations in peripheral input, as in the visual system. The general consequences of cochlear removal include degeneration of auditory nuclei in the brainstem [14, 15] and reorganization of axonal connectivity between the nuclei [16, 17]. Moreover, cochlear ablation may also result in various cellular and molecular changes, including those in gene expression, synaptic activity, and protein synthesis. For example, upregulation of growth associated protein- (GAP-) 43 and synaptophysin seems to indicate that neural circuits are subject to synaptic reorganization [18–20]. At the same time, some neurotrophins, such as insulin-like growth factor 1, have been identified that may contribute to synaptogenesis [19, 21]. All of these changes may contribute directly or indirectly to altered auditory pathway activity.

Several studies have demonstrated that unilateral cochlear ablation can enhance the responsiveness of neurons in the central auditory system. In cochlea-ablated neonatal gerbils, stimulation to the nonoperated side resulted in lower response thresholds, greater peak discharge rates, and reduced minimum response latency in inferior colliculus (IC) neurons, despite response patterns comparable to those of normal animals [22]. These changes are consistent with an increased proportion of excited neurons in the IC [23–25]. Measured with whole-cell voltage-clamp recordings in brain slices, the amplitude and duration of evoked excitatory postsynaptic currents (EPSCs) increased significantly, combined with a decrease in inhibitory postsynaptic current (IPSC) conductance and a depolarization of the IPSC reversal potential [26]. Similar electrophysiological results in the auditory cortex (AC) confirmed increased excitation after cochlear ablation [27, 28]. The mechanism underlying this enhanced performance was hypothesized to be a loss or downregulation of inhibitory influence [24]. Based on the time course of events, Mossop et al. [24] suggested two possible causes of the altered central response. First, functional unmasking, a stimulus-related phenomenon, may lead to an increase in responsiveness within minutes or hours. Deactivation of surrounding inhibitory circuits, which are normally used to suppress the response of the auditory pathway, intensifies excitatory inputs and increases overall responsiveness. Another possibility involves a delayed reduction in neurotransmitter-mediated inhibition. For example, GABAergic-associated events may be decreased, which may help explain long-term changes. The expression of GABA receptors and the level of GABA synthetase are both greatly compromised after cochlear ablation [24, 29]. Furthermore, GABA release was found to be elevated for a couple of days, but then it dropped over the long term. Moreover, the results varied to some extent at the lower levels of the brainstem [30]. This may indicate that changes in transmitters occur in a complex, dynamic manner that may set up chain reactions of downstream events. If GABAergic activity is related to hyperexcitability in the IC, other synaptic transmitters may also be involved, such as glutamatergic synapses. Cochlear ablation can result in upregulation of the expression of the glutamate receptor and levels of glutamate, as well as larger and longer NMDA receptor-mediated currents in auditory brainstem and cortex neurons [27, 31, 32]. Given that most phenomena of hyperexcitability are found in animals deafened neonatally, considering the significance of age and development, adult ablation attempts have been made to assess variation due to age. Discrepancies do exist. No sign of significant sound-evoked excitation in the IC was found in adult animals after cochlear ablation [23, 33]. McAlpine [34] described contradictory results, showing a dramatic increase in the proportion of IC neurons excited by the intact ear in adult animals, although cochlear removal in infancy resulted in a larger increase in the responsiveness of individual neurons than did the same treatment in adult animals. Moreover, there is evidence that the AC exhibits higher responsiveness than the IC, and neonatal deafening at later ages produced greater effects on the AC than on the IC [35]. The question of whether there are age differences in neural responses to auditory deprivation needs to be explored further.

## 3. Partial Lesions of the Cochlea

The organ of Corti functions as a receptor to interpret sounds received and to transform them into electrical signals. Hair cells are the main elements in the organ of Corti that participate in this process. It is not difficult to understand that any external damage that leads to certain pathological alterations in the hair cells would impair signal transmission and therefore lead to a series of changes in the central auditory circuits. Even minor injuries to subcellular structures within the organ of Corti should be considered.

### 3.1. Outer Hair Cells (OHCs)

Cisplatin is known to have ototoxic side effects, acting especially on the organ of Corti. Hair cells are the primary target, especially OHCs. Tinnitus is a common consequence of cisplatin chemotherapy. Increased spontaneous activity in the dorsal cochlear nucleus (DCN) was shown to be a contributing factor in the etiology of tinnitus [6]. Cisplatin-treated hamsters display enhanced spontaneous activity within the DCN associated with the loss of OHCs, particularly in the high-frequency region [36–38]. Furthermore, hyperactivity in the DCN was correlated with the degree of OHC loss [36–38]. These results suggest that OHC loss may be a primary initiator in a series of events leading to tinnitus. Evidence based on clinical cases corroborates this hypothesis because patients suffering from tinnitus exhibit significantly lower amplitudes of otoacoustic emissions (generating from OHCs) than do persons without tinnitus [39]. Thus, it is assumed that reduced OHC activity is related to the generation of tinnitus.

### 3.2. Inner Hair Cells (IHCs)

Compared with cisplatin, carboplatin preferentially damages inner hair cells (IHCs) at relatively low doses [40] and has been characterized as a selective IHC loss toxin [32, 41–44]. Changes associated with IHC loss manifest as a significant reduction in the compound action potential (CAP) [41, 43], which reflects the summed neural output across the total population of auditory nerve fibers. Moreover, the amount of reduction is proportional to the extent of IHC loss [43]. However, a decline in output from inner hair cells does not result in a dramatic reduction in inferior colliculus potential (ICP) or auditory cortex potential (ACP) [43]. In some cases, ACP amplitudes remained unchanged and were even higher than those in the pre-carboplatin-treated group [43]. These results are also supported by examinations of neuronal properties in the IC, which showed no threshold or tuning curve shifts and no significant difference in potential amplitudes [43, 45, 46]. Both transient and sustained enhancement of AC potential have been observed, indicating that cortical circuits are involved in the process [43]. Taking the reduced cochlear output into account, even unchanged AC or IC neuronal activities are considered to provide increased gain in the auditory pathway. The degree of nonmonotonic rate-level functions (RLFs) is decreased in the IC following carboplatin treatment, indicating reduced inhibition in the IC [44].

### 3.3. Ribbon Synapses

Ribbon synapses are responsible for synchronous auditory signaling and transmitter release, and they have been implicated in temporal resolution [47–50]. It has been suggested that these synapses play an important role in sound coding. Recent findings show that ribbon synapses may be the primary target of low-dose gentamicin treatment without overt morphological disruptions [51]. IHC ribbon loss can also be a result of a mild level of noise exposure [52, 53]. Behaviorally tested tinnitus was associated with the loss of IHC ribbon synapses due to deafferentation [54, 55]. Additionally, Arc, an immediate early gene encoding activity-regulated cytoskeletal protein, which is involved in synapse scaling, was reduced in the AC as a result of ribbon loss [55]. Arc has been demonstrated to be upregulated in the brain under sensory-enriched conditions [56], suggesting reduced Arc levels as a correlate of deafferentation, consistent with results in the periphery. Moreover, in Arc knockout mice, sensory experiences can reduce the ability to scale down excitatory synapses [57]. The notion that IHC ribbon loss may cause synaptic scaling plasticity, particularly a more excited profile, in the central auditory pathway is worthy of examination in future studies.

## 4. Combined and Incomplete IHC and OHC Injuries (Acoustic Trauma)

Noise exposure is known to result in insults to hair cells (IHCs and OHCs, OHCs preferentially), impaired hearing sensitivity, and elevated hearing thresholds. Although it is likely that the characteristics of the noise, such as the frequency, intensity, and duration, determine the varying patterns of these effects, an overview is still of great value. Changes in the properties of neurons following noise-induced hearing impairment are often regarded as the mechanism underlying dysfunction of the integrated and sophisticated central auditory system. There has been much research regarding neuronal hyperactivity in recent decades. High-intensity acoustic overstimulation (e.g., 2.8 kHz, 105 dB SPL, and 2 h) substantially reduced the amplitude of CAP and increased the hearing threshold by about 15 dB at 1 kHz compared with a preexposure group [41], indicating that the gross output of the cochlea declines due to a loss of sensory cells. Moreover, the input of the cochlear nucleus (CN) (approximately the output of the cochlea) exhibits a similar pattern to CAP [41]. However, the local field potential of IC is reduced at low intensities but shows a rapid increase at higher intensities, finally exceeding the level before noise exposure [41]. In addition, the lowered CAP threshold and spontaneous firing rates in the IC seem to be correlated. A study by Mulders et al. showed that the spontaneous firing rate of IC neurons was in direct proportion to the degree of hearing loss; that is, the more severe the hearing loss was, the greater the increase in the spontaneous firing rate was [58]. Hyperactivity in the IC is also evidenced by increased spontaneous firing rates and high incidences of burst firing [59, 60]. This kind of hyperactivity has been found at different levels of the auditory pathway to varying degrees, such as in the ventral cochlear nucleus (VCN) [8, 61, 62], DCN [63–66], and AC [67–70]. For example, compared with the lower response amplitudes of the auditory brainstem after noise exposure, auditory middle latency response (MLR) amplitudes and the slopes of MLR amplitude intensity function were increased [71]. Because MLR is generated from a higher level of auditory pathways than ABR, it is considered that ABR suppression reflects noise-induced alterations at the periphery, whereas MLR enhancement indicates increased responding status in the central auditory system [71]. The underlying relationship between hyperactivity in the IC and DCN has drawn considerable interest. Resection of the DCN does not abolish behavioral signs of tinnitus [72]. A possible explanation is that a superior level of the central auditory pathway seems to take part in the pathological process. An immediate and significant elevation of spontaneous firing rates is observed in DCN and VCN after noise trauma, whereas IC activity remains unchanged. However, 2 weeks after exposure, increased IC activity begins to be detected, along with continuous hyperexcitation in the DCN [62]. Delayed IC hyperactivity may be a result of progressive influence of DCN hyperactivity on the higher level [72]. To further determine the relationship with cochlear activity, cochlear ablations have been manipulated at different periods after acoustic trauma. IC hyperactivity can be stopped by afferent drive blocking before 8 weeks after exposure, whereas, after this time period, cochlear ablation has no effect, and the neurons become endogenously excited independent of cochlear input [7, 8, 73]. These data suggest that there is progressive centralization of hyperactivity in the IC and a “window phase” (around 8 weeks) in the process [73]. Moreover, it is possible that the DCN serves to convey excitation to the IC [74]. Manzoor et al. [75] compared electrophysiological characteristics of noise-induced hyperactivity in the two nuclei and found that hyperactivity showed similar time courses and tonotopic patterns, although spontaneous activity was much lower in the IC than in the DCN.

One possible explanation for the enhanced activity in the auditory nuclei is that the profile of inhibition weakens after acoustic trauma [76]. For example, dendrites in the posterior ventral cochlear nucleus (PVCN) suffer a net loss of both excitatory and inhibitory endings at first; later, the net number of excitatory endings recovers greatly, whereas the inhibitory terminals recover only partially [77]. However, in the DCN, inhibitory neurons are far more dominant than excitatory neurons, compared with the VCN [78]. It is supposed that the loss of inhibitory synapses with acoustic trauma is greater in the DCN than in the VCN, and this may be a major reason that the DCN rather than the VCN initiates these excitatory events. The neurotransmitter system may be responsible for mediating this process. For example, a decrease in GABAergic inhibition in the DCN was found in mice with behavioral evidence of tinnitus [6]. Enhanced evoked responses in the DCN are found in noise-induced tinnitus mice as well. Moreover, blocking GABAergic synapses greatly enhanced the evoked response in control mice versus that in tinnitus mice, with blocking excitation slightly decreasing responses in tinnitus mice. This conclusion is supported by parallel experiments on the IC showing that a GABA antagonist does not cause significant changes in the temporal integration of noise-exposed animals [79]. Measurement of inhibitory receptor-related mRNA (GABA-A receptor subunit alpha 1, GABRA1, and glycine receptor subunit alpha 1) expression revealed a comparable trend, decreasing first and then increasing later [80], indicating that gene expression regulates the reduction in inhibition. Moreover, the localized region of reduced GABRA1 expression corresponds to the region where hyperactivity of IC neurons has been shown to develop [81]. An unmasking model has also been suggested as an explanation for hyperactivity after acoustic overstimulation [41, 76], consistent with cochlear ablation [24]. Moreover, Wang et al. further investigated the role of disinhibition after acoustic trauma and suggested that the inhibition may help sharpen the tuning curve and hold the excitatory responses within a narrow range [76]. Correlatively, disinhibition may expand the excitatory response and increase neuronal discharge rates, thus creating an overexcited profile.

## 5. Models for Reference

There are many models and results that can be used for comparison. Aminoglycoside antibiotics, known for causing “classical” ototoxicity, were used frequently in models of deafness in early research. Studies with this model found increased evoked c-Fos expression, indicating enhanced neuronal activity, and a marked decrease in GABA release from the central nucleus of the IC [82]. Due to extensive damage to the organ of Corti by aminoglycoside antibiotics, the interpretation of links between the lesion sites and these changes is limited.

Presbycusis, hearing loss as a consequence of aging, is characterized by a loss of hair cells, and downregulation of inhibition, both glycinergic and GABAergic, is found in the CN [83, 84], IC [85], and AC [85]. However, despite widespread reduction in inhibition, excitability of neurons in the IC shows little change [85]. Some neuroscientists favor the view that age-related changes in the IC have no obvious link to peripheral hearing loss. Those changes that are related to a decline in temporal processing seem to be due to aging in the auditory brainstem, somewhat independent of the peripheral deficits [86]. Because degenerative changes appear to occur in the brain with age, it is not easy to determine whether the peripheral deficits result in or are responses due to the central changes. Perhaps these are not mutually exclusive; both may occur to some extent. Clinicians have indicated that tinnitus often occurs together with presbycusis, especially in those with more severe degeneration of outer hair cells and stria vascularis [87]. Do hair cells have nothing to do with the brain? So far, the relationship between downregulation of inhibition and hearing loss and changes in the neuronal properties of the aged brain are unclear and require further examination.

Recently, an alternative model has been developed with salicylate administration and confirmed in humans [88]. Salicylate has been shown to increase both spontaneous and stimulus-driven activity widely across the circuits [89–94], extending such effects, to some extent, to acoustic trauma [92]. Moreover, downregulation of GABA-mediated inhibition has also been observed [93, 95]. OHCs appear to be the peripheral lesion site, inducing the subsequent consequences in the central system [88]. The relationship between the peripheral and central systems is still unclear. Moreover, instead of systemic application, local application of salicylate in the cochlea [93] and directly in the IC [89] and AC [94] suggests that induced hyperactivity originates in central pathways rather than in the cochlea. It is possible that some cochlear traumas, particularly those related to hair cell deficits, lead to increased excitation in the central auditory system as a result of unmasking of excitation or downregulation of inhibition, which may underpin tinnitus [96].

## 6. Homeostatic Plasticity and Other Potential Mechanisms

As the suggested mechanisms vary, no firm conclusion can yet be drawn. One of the most inclusive and plausible explanations is the theory of homeostatic plasticity, briefly, the ability of a neuronal network to maintain its present state [97]. When the central system detects reduced input from the cochlea, homeostatic compensation occurs, intensifying the intrinsic activity of neurons to maintain the mean firing rates unchanged in the circuits, as depicted in a computational model [5]. It can also be assumed that appropriate additional acoustic stimulation may reverse such hyperactivity [5]. This assumption is supported by the fact that tinnitus can be reversed by providing an enriched environment that matches the impaired frequencies [98, 99] or by repeatedly pairing tones with brief pulses of vagus nerve stimulation [100]. In addition, cross-model reorganization, realized years ago, seems to fit well with homeostatic compensation theory and may play a role in trauma-exposed auditory plasticity. For example, DCN responses have been found to be enhanced with trigeminal stimulation following a noise-induced reduction in auditory nerve inputs [101], as both auditory and somatosensory stimuli converge in the DCN. Although somatosensory input normally has a suppressive effect on DCN responding, long-term somatosensory stimuli in noise-exposed animals surprisingly reverse this suppression effect, especially in animals with tinnitus [102]. Furthermore, these cross-modal effects are considered to be widely distributed because a large proportion of neurons with somatosensory inputs are found to be vigorously active across the core auditory cortex without auditory stimulation [103].

An imbalance in synaptic strength represents another possible mechanism, as cochlear damage may be followed by downregulation of inhibition. Basically, this refers to the tendency of a neuronal network to stabilize the total synaptic strength [5, 97]. With downregulation of inhibition, excitation may scale up as a response. Inhibition seems, at least in part, more susceptible to plasticity than excitation is. In the domain of normal hearing, both inhibitory and excitatory transmission function; by contrast, in the domain of impaired hearing, inhibitory synaptic efficiency decreases [104]. Considering that a balance can be achieved by increasing inhibition or by decreasing excitation, both have been tested, with results indicating that enhanced inhibition, rather than reduced excitation, reverses tinnitus behavior [104]. Accordingly, if inhibitory strength is truly compromised, targeting inhibitory strength may offer potential for reversing or alleviating neuronal hyperactivity [105]. GABA receptor agonists were shown to reverse tone-exposure-induced hyperexcitability in the IC of rats [106] and to relieve tinnitus in humans [107]. It is clear that decreased inhibition is involved in tinnitus-related plasticity, but the extent of this involvement remains unclear. Thus, the question of whether reducing inhibition can induce or increase neural hyperactivity is worthy of further confirmation.

Recently, much importance has been attached to the role of ion channels in the pathology of the hyperactive auditory brain. Researchers have found that exposure to excessive noise causes reduced activity in the voltage-gated potassium channel Kv7, which induces DCN hyperactivity and leads to the development of tinnitus. Manipulations that increase Kv7 activity or reduce the activity of another type of voltage-gated potassium channel, the HCN channel, can help to prevent tinnitus-associated hyperactivity [108, 109]. The changes in ion channel activity may collaboratively contribute to the neuronal hyperactivity induced by noise exposure. To date, theories of homeostatic plasticity and the roles of these ion channels in the development of neural hyperactivity have been built on noise-exposed animal models, which need to be studied further in the other cochlear damage models.

## 7. Concluding Remarks

Based on the research reviewed, it seems likely that specific insults to the peripheral auditory system, including cochlear ablation, selective IHC or OHC loss, and noise-induced mixed and incomplete IHC and OHC injuries, result in a reduction of input from the cochlea, thereby giving rise to hyperactivity in the central auditory circuits. A good example of this process is found in tinnitus, which may be associated with neuronal hyperactivity and is likely a common consequence of various kinds of cochlear damage. From an evolutionary perspective, hyperactivity in the brain may be a maladaptive response to reduced input, indicating that the system needs to become more sensitive to the reduced input to obtain more information and thereby remain balanced and stable. This dysfunctional neural state might contribute to some brain pathologies with auditory dysfunction, as indicated in a recent review suggesting that hyperactivity in the auditory brain is closely related to tinnitus and hyperacusis [110]. Despite these findings, it is still too early to say that hyperactivity in the auditory brain follows cochlear damage. Hopefully, a better understanding of altered neural properties in response to cochlear damage will provide new insights into the mechanism of injury-induced central plasticity, suggesting novel strategies for therapies.
